# Adjunctive PC6 magnetic stimulation with rTMS over left DLPFC for post-stroke cognitive impairment: a protocol for fNIRS-fMRI randomized controlled trial

**DOI:** 10.3389/fneur.2026.1714491

**Published:** 2026-04-09

**Authors:** Ning Sun, Yi He, Yi-Wei Liu, Gang-Lin Chen, Lei Chen, Hui-Lin Yang, Jing-Kang Lu, Jing Xiong, Xiao-Yi Xiong, Cheng-Qi He

**Affiliations:** 1 Rehabilitation Medicine Center and Institute of Rehabilitation Medicine, West China Hospital, Sichuan University, Chengdu, China; 2 Key Laboratory of Rehabilitation Medicine in Sichuan Province, West China Hospital, Sichuan University, Chengdu, China; 3 Acupuncture and Tuina School, Chengdu University of Traditional Chinese Medicine, Chengdu, China; 4 Department of Rehabilitation Medicine, West China Tianfu Hospital, Sichuan University, Chengdu, China; 5 Sichuan Provincial Key Laboratory for Acupuncture & Chronobiology, Chengdu, China; 6 Key Laboratory of Acupuncture for Senile Disease (Chengdu University of TCM), Ministry of Education, Chengdu, China

**Keywords:** acupoint magnetic stimulation, PC6 acupoint, post-stroke cognitive impairment, randomized controlled trial, study protocol

## Abstract

**Background:**

Post-stroke cognitive impairment (PSCI) is a common stroke complication, significantly reducing patients’ quality of life and increasing caregiving burden. Repetitive transcranial magnetic stimulation (rTMS) of the left dorsolateral prefrontal cortex (L-DLPFC) improves cognition but with limited efficacy. Stimulation of Neiguan (PC6), a key acupoint in traditional Chinese medicine, modulates cognition-related brain networks.

**Objective:**

To compare cognitive improvement and brain network remodeling in PSCI patients who receive rTMS over the L-DLPFC combined with (1) PC6 magnetic stimulation (MAG), (2) PC6 acupuncture (ACU), or (3) PC6 sham magnetic stimulation (SHM).

**Methods:**

This three-arm randomized controlled trial will enroll 105 patients with PSCI, randomly allocated 1:1:1 to the MAG (*n* = 35), ACU (*n* = 35), and SHM (*n* = 35) groups. Interventions will be administered 5 times/week for 3 weeks. All groups will receive high-frequency rTMS over the L-DLPFC (10 Hz, 80% rest motor threshold). The primary outcome is the Montreal Cognitive Assessment (MoCA) change from baseline to week 3. Secondary outcomes include the Mini-Mental State Examination (MMSE), Modified Barthel Index (MBI), Hamilton Anxiety Rating Scale (HAMA), Hamilton Depression Rating Scale (HAMD), Pittsburgh Sleep Quality Index (PSQI), and adverse events. Functional magnetic resonance imaging (fMRI) and functional near-infrared spectroscopy (fNIRS) data will be acquired at baseline and post 3-week treatment. Clinical data will be analyzed via SPSS 26.0 using repeated measures analysis of variance (ANOVA) to compare intergroup changes in outcomes across time points. Neuroimaging data will be processed in MATLAB R2018a. Correlation analyses will assess associations between clinical scores and neuroimaging parameters.

**Conclusion:**

This study will provide the first randomized controlled evidence for acupoint magnetic stimulation in PSCI, demonstrating that adding MAG to L-DLPFC rTMS confers additional cognitive benefits. Its mechanism in reshaping brain networks will be elucidated via fNIRS-fMRI, which is expected to accelerate clinical translation of non-pharmacological interventions.

**Clinical trial registration:**

https://www.chictr.org.cn/, identifier ChiCTR2400090768.

## Introduction

Post-stroke cognitive impairment (PSCI) is one of the common sequelae in stroke survivors (1). Globally, the incidence of PSCI varies due to regional differences and diagnostic criteria, with approximately one-third to one-half of stroke patients demonstrating cognitive impairment during follow-up (2). PSCI not only reduces patients’ ability to perform activities of daily living but also significantly increases disability and mortality rates, imposing a heavy burden on families and society (3). China is one of the countries bearing the greatest global burden of stroke, both in terms of incidence and disease impact (4). Currently, no pharmacological therapy has proved universally effective for PSCI, making non-pharmacological interventions a key focus of research (5).

Cognitive deficits following stroke are linked to large-scale network dysfunctions, including disrupted frontotemporal connectivity within and between the default-mode networks (DMN), salience networks (SN), and central executive networks (CEN) (6–9). High-frequency repetitive transcranial magnetic stimulation (rTMS) targeting the left dorsolateral prefrontal cortex (L-DLPFC) may be the most promising approach for improving overall cognitive function after stroke, with no significant safety concerns (10). Targeted stimulation of the DLPFC, an essential hub for working memory, attention, and executive control, may enhance cortical excitability and thereby underlie its therapeutic benefit (11). Building on central stimulation, repetitive peripheral magnetic stimulation (rPMS) delivers high-intensity magnetic pulses to peripheral nerves, inducing proprioceptive afference that up-regulates sensorimotor cortices and mitigates motor-cognitive deficits (12, 13).

In addition to magnetic neuromodulation, acupuncture as a cornerstone of traditional Chinese medicine (TCM) has been widely used in cognitive rehabilitation. Endorsed by the World Health Organization (WHO) in 2002 as an adjunctive therapy for vascular dementia (14), acupuncture therapy (manual, electro-, and moxibustion) have repeatedly improved cognitive outcomes (15). Among acupoints, Neiguan (PC6) is a primary target for cognitive disorders (16). Functional magnetic resonance imaging (fMRI) reveals that PC6 stimulation modulates vestibulo-autonomic regions (cerebellar flocculonodular lobe, hypothalamus, insula) and key cognitive networks such as the default-mode and anterior cingulate cortices (17, 18). In recent years, research teams have attempted to apply modern magnetic stimulation techniques to traditional acupoints. Recent studies have applied magnetic stimulation to PC6, reporting enhanced small-world properties and interregional synchrony within cognitive networks, thereby facilitating information transfer (19, 20). These findings imply a dual mechanism: classical meridian regulation combined with direct neuromodulation, potentially yielding adjunctive cognitive benefits.

Advances in neuroimaging enable multidimensional tracking of post-stroke brain reorganization. Functional near-infrared spectroscopy (fNIRS) and fMRI are the principal tools. fNIRS provides portable, bedside-capable monitoring of cortical hemodynamics (21), with studies revealing reduced inter-hemispheric connectivity in PSCI patients compared to controls (22). Resting-state fMRI, by mapping spontaneous BOLD synchrony, excels at delineating whole-brain functional network integrity and its disruption in PSCI (23, 24). Integrating fNIRS, fMRI, and other modalities merges functional and structural data, offering a comprehensive view of network states and an objective means of evaluating intervention efficacy.

This prospective randomized controlled trial (RCT) will assess whether magnetic stimulation at PC6 improves cognition in PSCI and clarifies its neural basis. We hypothesize that, compared to the control intervention, the PC6 magnetic stimulation will significantly improve patients’ overall cognitive level and exhibit specific neuroplastic changes in brain functional networks. As the first multimodal study integrating acupoint therapy with neuromodulation, this research will provide new insights and high-quality evidence for non-pharmacological management of PSCI.

## Method and analysis

### Research design

**Figure 1:**
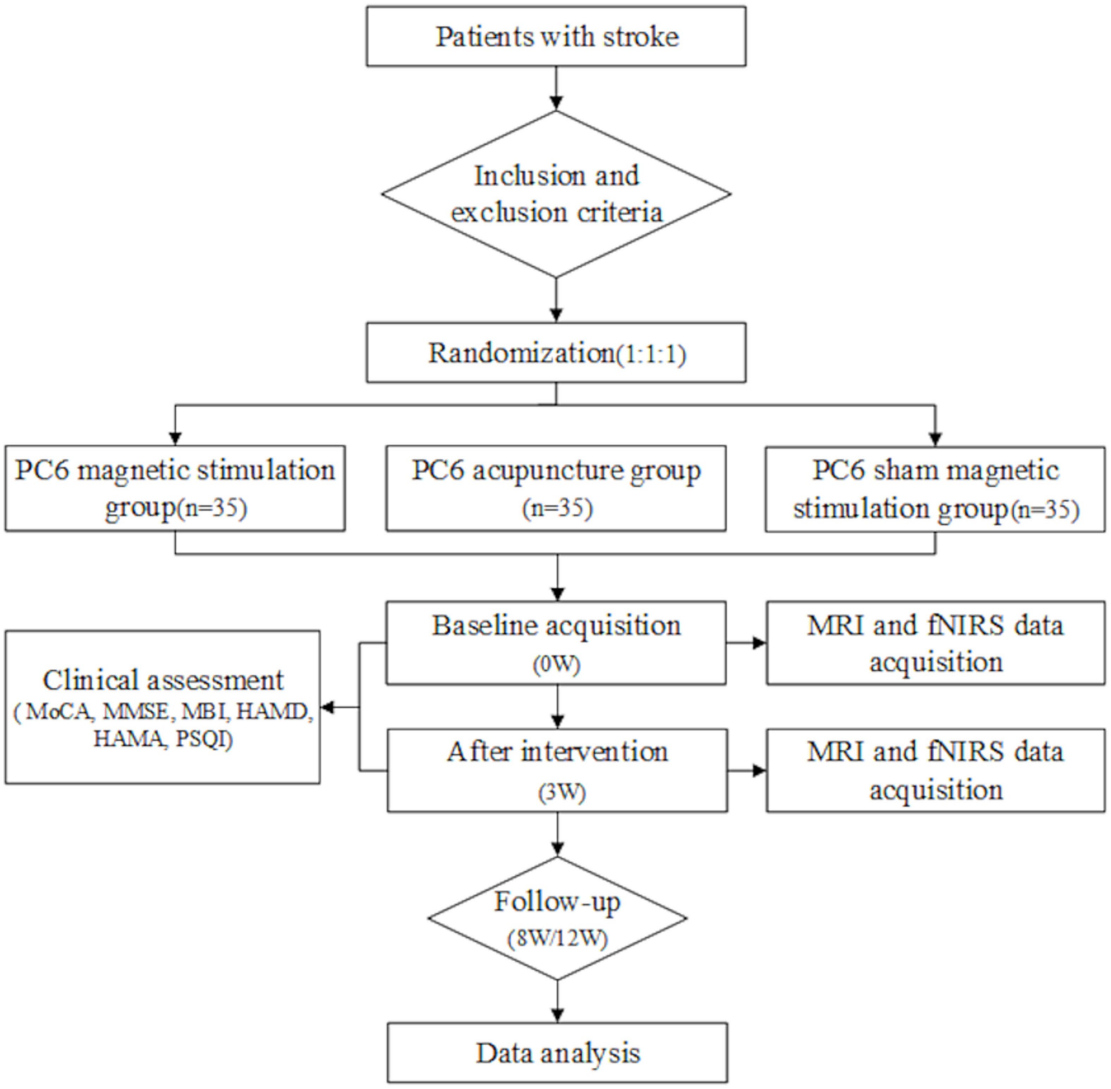
Trial flow chart. MoCA, Montreal Cognitive Assessment; MMSE, Mini-Mental State Examination; MBI, Modified Barthel Index; HAMD, Hamilton Depression Rating Scale; HAMA, Hamilton Anxiety Rating Scale; PSQI, Pittsburgh Sleep Quality Index.

The present study is a three-arm RCT. Eligible PSCI patients will be enrolled from the Inpatient Department and Outpatient Clinic of Rehabilitation Medicine, West China Hospital, Sichuan University. Participants will be randomized in a 1:1:1 ratio to three groups: PC6 Magnetic Stimulation (MAG), PC6 Acupuncture (ACU), and Sham Magnetic Stimulation at PC6 (SHM). All three groups will concurrently receive rTMS (targeting the L-DLPFC). The study flowchart is presented in Figure 1, and assessment time points are detailed in Table 1. Ethical approval was granted by the Ethics Committee of West China Hospital of Sichuan University on March 13, 2024 (Approval No.: 20240313). The trial is registered at the Chinese Clinical Trial Registry: https://www.chictr.org.cn/, Registration No.: ChiCTR2400090768.

**Table 1:** Details of the planned visit schedule.

Stage	Baseline period	Treatment period			Follow-up period	
TimePoint	0	Week 1	Week 2	Week 3	Week 8	Week 12
Enrollment						
Eligibility screen	×					
Informed consent	×					
Demographic characteristics	×					
Medical history	×					
fNIRS	×			×		
fMRI	×			×		
Interventions						
Magnetic Stimulation Group		×	×	×		
Acupuncture Group		×	×	×		
Sham Magnetic Stimulation Group		×	×	×		
Assessments						
MoCA	×			×	×	×
MMSE	×			×	×	×
MBI	×			×		
HAMA	×			×		
HAMD	×			×		
PSQI	×			×		
Patient safety						
Adverse events		×	×	×		

### Diagnostic criteria

Refer to the diagnostic criteria for PSCI in the Scientific Statement on Cognitive Impairment After Ischemic and Hemorrhagic Stroke (25), published by the American Heart Association/American Stroke Association (AHA/ASA) in 2023.

### Inclusion criteria

1. Aged 30–75 years, right-handed;
2. Definite history of stroke, with clinical and imaging evidence supporting the diagnosis;
3. Presence of impairment in one or more cognitive domains, with a MoCA score <26;
4. Stable vital signs;
5. Written informed consent from the patient or legal guardian.

### Exclusion criteria

1. Unstable vital signs or uncontrolled severe respiratory/circulatory complications;
2. Recurrent stroke or multiple lesion strokes;
3. Pre-existing epilepsy, cognitive impairment, depression, other psychiatric disorders;
4. Secondary cognitive impairment (e.g., excessive alcohol consumption);
5. Severe dysfunction of other organs or critical systemic diseases;
6. Severe visual/hearing impairments or aphasia precluding cooperation;
7. Neurodegenerative cognitive disorders (e.g., frontotemporal dementia);
8. Contraindications for rTMS treatment and MRI examination: Metallic implants/electronic devices, skull defects, etc.

### Withdrawal criteria

A participant will be withdrawn from the trial if any of the following conditions occur:

1. Serious adverse reactions requiring termination, as determined by a specialist physician;
2. Worsening symptoms or severe complications necessitating emergency measures;
3. Identification of critical safety concerns by investigators;
4. Voluntary withdrawal for personal, family, or social reasons that precludes completion of the treatment course.

### Sample size

This study employes a three-arm, parallel-group, randomized controlled design (1:1:1 allocation), with the MoCA score as the primary endpoint. One-way analysis of variance (ANOVA) is used to compare differences among the three groups. Due to the lack of a clear anticipated effect size, based on previous rTMS studies in PSCI (26–28), we adopt Cohen’s f ≈ 0.32 as the expected effect size. Using G*Power 3.1 (test type: one-way ANOVA; effect size *f* = 0.32; *α* = 0.05, two-tailed; power = 0.80), the estimated total sample size is 99 (33 per group) to detect significant differences in MoCA scores. To account for a potential 10% attrition rate, the final recruitment target is set at 105 participants (35 per group). Because *a priori* information on within-subject correlation and covariate R^2^ was unavailable, we used one-way ANOVA for conservative planning. The primary analysis will use longitudinal models with covariate adjustment, which is expected to provide equal or greater efficiency than the planning model.

### Randomization and blinding

The allocation sequence is computer-generated by an independent statistician using block randomization (block size = 3) with a 1:1:1 ratio for the MAG, ACU, and SHM group. Allocation concealment is implemented using sequentially numbered, opaque, sealed envelopes. Each envelope contained an allocation card corresponding to the group assignment. Envelopes will be opened sequentially by treating physicians to deliver the allocated intervention.

Given the nature of the magnetic stimulation, acupuncture, and sham magnetic stimulation intervention therapies, it is not possible for the treating physicians to be blinded in this trial. However, participants, outcome assessors, and data analysts will be blinded to group allocation. To minimize bias, strict separation of roles is maintained between intervention operators, outcome assessors, and statisticians throughout the trial. Blinding assessment will be conducted after the final treatment session. All participants will be asked to guess their group allocation from four options: PC6 MAG, PC6 ACU, PC6 SHM, or uncertain. These responses will be used to calculate the James Blinding Index, which quantifies blinding success on a scale from 0 to 1, where 1 indicates complete blinding (all “do not know”), 0 indicates complete unblinding (all correct), and 0.5 represents random guessing (29). Given the potentially perceptible differences between active magnetic stimulation and sham stimulation, a higher risk of functional unblinding may exist between the MAG and SHM groups, which will be considered when interpreting the blinding assessment results.

### Intervention and grouping

**Figure 2:**
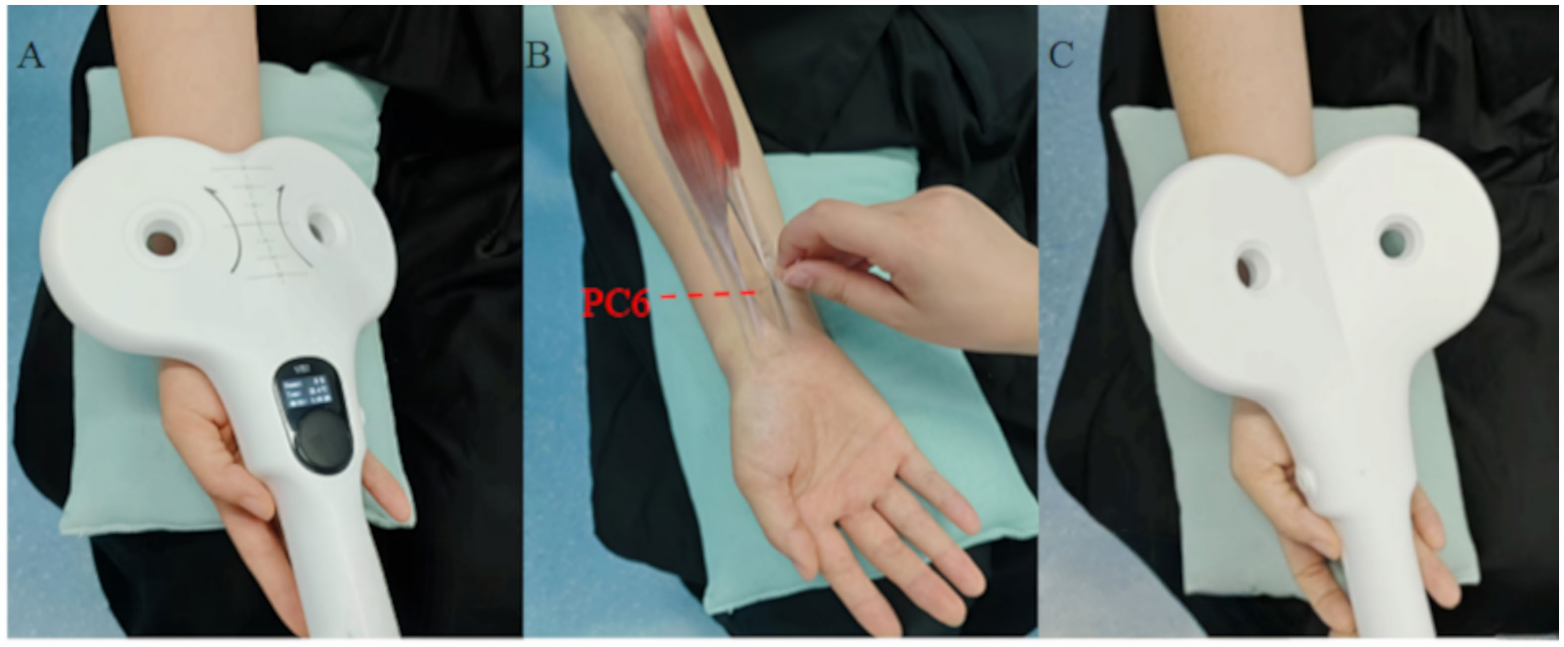
Three specific intervention methods. **(A)**, PC6 magnetic stimulation; **(B)**, PC6 localization and acupuncture at PC6; **(C)**, PC6 sham magnetic stimulation.

PSCI patients will be randomly assigned to the MAG, ACU, or SHM group. The stimulation target in all groups is the PC6 acupoint (unilateral, right side), located according to the WHO Standard Acupuncture Point Locations on the anterior forearm, between the tendons of the palmaris longus and flexor carpi radialis, approximately 2 B-cun proximal to the palmar wrist crease (Figure 2B). Each group will receive 15 treatment sessions (once daily, 5 days per week for three consecutive weeks).

Across all groups, high-frequency rTMS will be administered over the L-DLPFC concurrently with each session of the assigned intervention. This protocol is designed to reflect the “central regulation + peripheral input” rehabilitation model and to ensure ethical consistency across treatment arms. In addition, all patients will receive conventional rehabilitation delivered by a multidisciplinary team in accordance with the Adult Stroke Rehabilitation Guidelines (NICE NG236) (30). This includes physical therapy, occupational therapy, and speech and language therapy, provided for at least 3 h per day, on at least 5 days per week, tailored to individual goals and needs. Rehabilitation also encompasses swallowing assessment and management, prevention of complications, and support for cognitive, psychological, and functional recovery, as outlined in the guideline recommendations for comprehensive stroke rehabilitation. We will document the type, frequency, and duration of conventional rehabilitation received by each participant. If a specific intervention within conventional rehabilitation is likely to influence a patient’s cognitive level, the investigator will document its type, duration, and intensity in detail in the Case Report Form form. Therefore, we will systematically document lesion side (left/right) and key lesion features (e.g., location and volume where available).

#### rTMS over L-DLPFC procedure

rTMS will be performed using a MagNeuroR470 magnetic stimulator (Nanjing Weisi Medical Technology Co., Ltd.), equipped with a double-cone coil and a figure-of-eight coil. The MAG group and SHM group will utilize a paired-pulse mode. For L-DLPFC localization, the “5-cm rule” (31) will be adopted, and the double-cone coil will be vertically positioned to cover the target cortex. To enhance targeting reproducibility and provide auditable documentation, we strengthened cap-based quality control and recording procedures: the target point will be identified on a fitted 10–20 cap and marked at baseline for replication across sessions; cap landmarks (nasion–inion and preauricular points) will be checked at each session; coil orientation/positioning will follow a predefined SOP; coil placement and deviations will be documented. In addition, we will record the target location relative to 10–20 landmarks (e.g., the marked scalp coordinate) to enable protocol adherence auditing across sessions and participants. To ensure consistency and reproducibility across all participants and sessions, all operators will undergo standardized training on coil positioning and stimulation procedures, and a strict, documented protocol will be followed for every stimulation session. Stimulation parameters: high frequency of 10 Hz, intensity of 80% of the resting motor threshold (RMT). RMT will be determined via stimulation of the abductor pollicis brevis muscle on the affected side. Each session will consist of 1,000–1,500 pulses.

##### RMT measurement

RMT will be measured before treatment, defined as the minimum TMS intensity that induces a motor evoked potential (MEP) with a peak amplitude greater than 50 μV in the target muscle at rest in at least 5 out of 10 consecutive trials (32). Subjects will be seated in a stable wooden chair and instructed to remain relaxed but awake throughout the procedure. Recording and reference electrodes will be attached to the muscle belly and tendon of the affected abductor pollicis brevis muscle, respectively, with the ground electrode attached to the dorsum of the ipsilateral hand; inter-electrode spacing will be approximately 2 cm. Patients will be fitted with a positioning cap designed according to the 10–20 electroencephalogram (EEG) system for assisted localization. The figure-of-eight coil will be tangentially fixed to the C3 position on the positioning cap, above the M1 motor area of the dominant hand. Single-pulse stimulation will be initiated at a higher intensity. When 5 out of 10 single-pulse stimulations elicit MEP amplitudes > 50 μV, the stimulator intensity at that point will be defined as RMT (33).

#### Group-specific interventions

##### MAG group

Patients will receive peripheral magnetic stimulation at the PC6 acupoint(Figure 2A). A figure-of-eight coil (outer diameter: 114.8 mm, inner diameter: 79.6 mm) will be used to deliver peripheral magnetic stimulation. The stimulation frequency will be 20 Hz (high frequency), with intensity gradually increased from 80% RMT until slight flexion of the index and middle fingers is observed. The total stimulation duration per session will be 24 min.

##### ACU group

Patients will receive manual acupuncture at the PC6 acupoint (Figure 2B). All acupuncture procedures will be performed by the same two acupuncturists with over 6 years of clinical experience, who have completed clinical operational training organized by the research team. Following routine disinfection of the local skin at the acupoint, sterile disposable stainless-steel filiform needles (0.25 mm × 40 mm) will be inserted. And lifting-thrusting and twirling techniques will be applied to achieve deqi. Needle manipulation will be performed once every 10 min to maintain needle sensation, with a total of two manipulations, and the needles will be retained for 30 min.

##### SHM group

Patients will receive sham peripheral magnetic stimulation at the PC6 acupoint (Figure 2C). A figure-of-eight coil will be used, with the stimulation frequency set to 0 Hz and intensity set to 0. The coil will be positioned with its non-active (reverse) side facing the skin to ensure that no magnetic field is delivered to the median nerve. However, the auditory output of the device will be identical to that of the active stimulation to maintain blinding (34). The stimulation procedure, including coil placement, duration, and pulse protocol structure, will be identical to that used in the MAG group.

### Outcomes

#### Primary outcome measurement

This study will use changes in Montreal Cognitive Assessment (MoCA) scores before and after treatment as the primary outcome measure. The MoCA is a comprehensive cognitive assessment tool that covers seven domains (visuospatial/executive function, naming, attention, language, abstraction, delayed recall, and orientation). The total score is 30, with a cutoff value of 26. A score below 26 indicates cognitive impairment, and the lower the score, the poorer the cognitive ability (35).

#### Secondary outcomes

Secondary endpoints comprise global cognition, functional independence, anxiety, depression, and sleep quality, each assessed with a validated instrument as detailed below.

1. Mini-Mental State Examination (MMSE): the 30-item MMSE assesses orientation, immediate recall, attention/calculation, delayed recall, language, and visuospatial ability. Education-adjusted cut-offs are ≤17 (illiterate), ≤20 (primary education), and ≤24 (≥junior-high); lower scores denote more severe impairment (36).
2. Modified Barthel Index (MBI): this scale is a specialized tool for evaluating activities of daily living (ADL), comprising 10 assessment items, including eating, bathing, personal hygiene, dressing, bowel and bladder control, bed-to-chair transfer, walking on level ground, wheelchair operation, and ascending/descending stairs. During the assessment, evaluators score each item based on the patient’s level of assistance required. Ten items yield a 0–100 score; lower scores indicate greater ADL dependence (37).
3. Hamilton Anxiety Rating Scale (HAMA): this scale contains 14 items, with 7 items each for somatic anxiety and psychic anxiety. Total scores ≥7 suggest anxiety; higher scores reflect increased severity (38).
4. 17-item Hamilton Depression Rating Scale (HAMD): this scale includes 17 items, covering dimensions such as anxiety/somatization, weight change, cognitive impairment, diurnal variation, retardation, sleep disturbances, and feelings of hopelessness. Total scores ≥7 indicate possible depression; higher scores correspond to greater severity (39).
5. Pittsburgh Sleep Quality Index (PSQI): the PSQI consists of 18 items, covering seven components: sleep onset latency, sleep duration, sleep efficiency, subjective sleep quality, sleep disturbances, use of hypnotic medications, and daytime dysfunction. The total score ranges from 0 to 21, with higher scores indicating poorer sleep quality (40).

#### Assessment time

At baseline and at the end of the intervention, all pre-specified outcome measures will be assessed. At the 8-week and 12-week follow-ups, only the MoCA and MMSE will be used.

### Safety assessment

Investigators will be required to monitor and document all adverse reactions in the case report forms (CRFs) according to predefined criteria.

### Imaging data acquisition

#### MRI data acquisition

All MRI examinations will be performed at the MRI Center of West China Hospital, Sichuan University, on a 3.0 T scanner (Discovery MR750; GE Healthcare, Milwaukee, USA) equipped with an eight-channel phased-array head coil. Before scanning, each participant will be screened for contraindications and asked to remove all metallic items. Foam cushions will be placed around the head to minimise motion, and earplugs will be provided to attenuate acoustic noise. Scans will be acquired with the participant lying supine and remaining alert throughout.

A high-resolution structural dataset will be obtained with a three-dimensional T1-weighted spoiled gradient-echo sequence: repetition time/echo time (TR/TE) = 6.2/2.5 ms, field of view = 256 × 256 mm^2^, matrix = 256 × 256, voxel size = 1.0 × 1.0 × 1.0 mm^3^. BOLD images will be acquired using a gradient echo planar imaging (GRE-EPI) sequence: TR/TE = 2000/30 ms, flip angle = 90 degrees, slice thickness = 2 mm, field of view (FOV) = 128 × 128 mm^2^, voxel size = 2 × 2 × 2 mm^3^. DTI sequences will be acquired FOV = 256 × 256 mm^2^, TR = 6,500 ms, matrix = 128 × 128, number of diffusion-encoding directions = 32, slice thickness 2 mm, layer spacing = 0, and gradient values b = 0 s/mm^2^ and b = 1,000 s/mm^2^.

#### fNIRS data acquisition

This study will employ fNIRS to repeatedly acquire patients’ brain functional activity at three time points (before the first treatment, after the first treatment, and after the last treatment). Data will be collected using an fNIRS system (NIRSCAN, Huichuang, China) with wavelengths of 740 nm and 850 nm, consisting of 48 effective channels (22 light source probes and 16 detector probes). Cortical hemodynamic changes will be sampled at 11 Hz, with 3 cm inter-channel spacing covering the frontal and parietal lobes.

During data acquisition, the assessment room will be kept quiet, with only the patient and examiner present. The patient will be seated upright with head immobilized, and the probe array will be secured to the scalp. After probe adjustment and verification of optimal signal quality across all channels, data collection will began. The Verbal Fluency Test (VFT) (41) will be used as the cognitive activation task. As a validated measure of executive function, VFT reflects complex cognitive processes and has been extensively used in fNIRS research (42). Each trial will comprise a 60-s pre-task baseline, a 60-s task period (divided into three 20-s blocks), and a 60-s post-task recovery period. During the task, participants will generate as many words as possible based on three randomly presented Chinese characters (e.g., ‘Jiang’, ‘Ri’, ‘Da’), with the number of valid words recorded as a measure of cognitive performance.

Based on potential factors such as patient intolerance to imaging procedures, imaging data quality issues, or other possible interfering factors, we anticipate an imaging attrition rate of approximately 10%.

### Objectivity in outcome assessment and quality control

All clinical outcomes will be assessed by trained evaluators who are blinded to group allocation and not involved in treatment delivery. Assessors will follow a standardized assessment manual to minimize inter-rater variability, and role separation will be maintained between intervention operators, outcome assessors, and statisticians. Data will be recorded on case report forms and verified by independent checking to reduce entry errors, and protocol deviations as well as concomitant rehabilitation potentially affecting cognition will be documented. For neuroimaging, preprocessing and metric extraction will follow a pre-specified pipeline, with imaging analysts blinded to group allocation. Quality control will include motion/artifact inspection and predefined exclusion criteria.

### Statistical analysis

#### Clinical data analysis

All analyses will be performed using SPSS 26.0 (IBM, Armonk, NY, USA) with two-tailed tests (*p* < 0.05). Quantitative data will be expressed as mean ± standard deviation. Within-group comparisons will use paired t-tests. Between-group comparisons will employ one-way ANOVA or nonparametric rank-sum tests (Kruskal-Wallis test), depending on data distribution. Count data will be presented as numbers (percentages), to be analyzed by *χ*^2^ tests, Fisher’s exact tests, or Ridit analysis. Repeated-measures ANOVA with Greenhouse–Geisser correction will be applied to evaluate longitudinal changes across timepoints. Both intention-to-treat (ITT) and per-protocol (PP) analyses will be conducted. In the ITT analysis, missing outcome data will be handled using multiple imputation under the assumption of missing at random. For the primary outcome (MoCA), we will report effect sizes and 95% confidence intervals for between-group differences in score changes. Clinical relevance will be interpreted using published MoCA change ranges and distribution-based benchmarks, rather than a prespecified minimal clinically important difference (MCID). Pearson’s or Spearman’s correlation coefficients (selected based on data distribution) will be calculated to examine associations between clinical scales and neuroimaging parameters. Covariates including age, sex, years of education, time from stroke onset to study enrollment, and a quantitative metric capturing the “dose” of conventional rehabilitation (derived from documented type, frequency, and duration), and lesion side (left/right) will be incorporated into a multivariate linear regression model evaluating the primary endpoint (change in MoCA score) and key secondary cognitive outcomes. Furthermore, to explore the potential modifying effect of lesion side on treatment response, we will conduct an exploratory analysis by adding a Group × Lesion Side interaction term to the primary multivariate model for the MoCA change score and key imaging outcomes. These interaction analyses will be explicitly labeled as exploratory, and their results will be interpreted cautiously due to the limited statistical power for detecting interaction effects.

### Neuroimaging data analysis

#### Primary and exploratory imaging outcomes

The following two measures are specified as the primary imaging outcomes: (1) functional connectivity (FC) of resting-state fMRI; and (2) prefrontal regions of interest (ROI) activation during the VFT as measured by fNIRS. These primary endpoints are considered confirmatory, with hypothesis testing guided by FDR correction as described below. All other imaging metrics described in this section are considered exploratory, and any observed associations will be interpreted as hypothesis-generating, requiring validation in future studies.

All neuroimaging data will be processed and analyzed using SPM12 (Wellcome Centre for Human Neuroimaging, London, UK), Homer2, and the NIRS-SPM toolbox in the MATLAB R2018a environment. To evaluate potential attrition bias, we will compare baseline demographic and clinical characteristics between participants who complete imaging assessments and those who do not. Furthermore, where feasible, we will consider using mixed-effects models to reduce bias associated with missing data. We will report reasons for missing imaging data.

#### fMRI data processing and analysis

BOLD-fMRI data preprocessing steps will include: slice timing, realignment, normalization, detrending, and bandpass filtering (0.01–0.08 Hz). Subsequently, the following brain functional indicators will be calculated: FC—the primary fMRI outcome—will be analyzed to assess network-level changes, while exploratory indicators will include amplitude of low-frequency fluctuation (ALFF), fractional ALFF (fALFF), regional homogeneity (ReHo), and functional connectivity strength (FCS). In addition, 3D-T1 structural images will be analyzed using SPM12 with the CAT12 toolbox for voxel-based morphometry (VBM) to extract structural metrics such as gray matter volume and cortical thickness. DTI data will be preprocessed using FSL 4.1.9 software, including head motion correction, eddy current correction, and tensor model fitting, to obtain fractional anisotropy (FA) and mean diffusivity (MD) maps.

#### fNIRS data processing and analysis

After format conversion, fNIRS raw data will undergo channel quality assessment, motion artifact correction (Wavelet-MDL), bandpass filtering, optical density change calculation, and HbO/HbR concentration extraction. The task paradigm will be a VFT, and a generalized linear model (GLM) will be constructed using an event-related design to extract the *β* value for each channel. Based on channel layout, ROIs such as the frontal lobe and motor areas will be defined. Activation within the prefrontal ROI during VFT—the primary fNIRS outcome—will be extracted as average activation intensities for statistical comparisons, including between-group and within-time point comparisons using ANOVA or nonparametric tests as appropriate. Repeated measures analysis of variance will be used to test Group × Time interaction effects. Exploratory analyses may include calculation of functional connectivity matrices to assess brain network integration and local clustering characteristics.

#### Multiple comparisons correction

For multiple comparisons correction across metrics and modalities, the following strategies will be applied: For primary imaging endpoints, FDR correction will be implemented to control for type I error—specifically, for fMRI functional connectivity analyses, FDR correction will be applied at the ROI and brain network level, and for fNIRS prefrontal ROI activation analyses, FDR correction will be applied at the channel level or ROI level, as appropriate. For secondary imaging metrics, appropriate correction methods will be applied within each set of analyses, and findings will be interpreted with caution.

## Discussion

This protocol describes a three-arm randomized controlled trial designed to determine whether PC6 magnetic stimulation provides additional cognitive benefit when combined with standard left DLPFC rTMS in patients with post-stroke cognitive impairment, and to characterize associated network-level remodeling using combined fNIRS-fMRI. By comparing PC6 magnetic stimulation with PC6 acupuncture and sham magnetic stimulation at PC6 while holding left DLPFC rTMS constant across arms, the study aims to quantify the incremental effect of PC6 magnetic stimulation versus sham and to compare magnetic stimulation with acupuncture. The trial’s multimodal imaging framework is expected to provide mechanistic insight into how this combined “central regulation and peripheral input” strategy may reshape cognition-related networks.

Cognitive impairment after stroke has been shown to be closely related to disruptions in multiple brain network connections. Resting-state functional MRI studies have found that patients with PSCI exhibit significantly reduced local functional connectivity in cognitive-related networks, including the DMN, SN, cerebellar network, and orbitofrontal cortex, with particularly prominent impairments in the functional connectivity and nodal efficiency of the bilateral caudate nuclei (43). In other words, stroke disrupts both local and global functional connectivity, particularly damaging information transmission pathways involved in cognition, and the extent of this network damage correlates closely with cognitive deficits (44, 45). A prospective study indicated that stronger interhemispheric connectivity of the DMN during the subacute phase of stroke predicts better cognitive recovery at 6 months; conversely, overactivation of the SN may indicate poorer cognitive outcomes (46). Therefore, enhancing the integration of cognitive brain networks in PSCI patients is considered a key approach to promoting cognitive rehabilitation (47). This study targets this mechanism through magnetic stimulation of the PC6 acupoint, located on the medial forearm near the distribution of the median nerve (48). We hypothesize that magnetic stimulation of this acupoint can activate widespread central networks via sensory afferents, thereby strengthening interactions among higher-order networks such as the DMN and CEN and restoring the disrupted network balance after stroke. Concurrently, high-frequency rTMS applied to the DLPFC focuses on enhancing the executive control network, and the combination of these two interventions is expected to jointly improve brain network function at different levels.

PC6 was chosen for its dual relevance in TCM and modern neuroimaging. In TCM, cognitive disorders reflect disturbance of ‘Shen’ (spirit), and PC6 is traditionally used to calm the mind and regulate cardio-cerebral function (49). This study combines this empirical knowledge with modern technology, precisely applying magnetic stimulation to PC6 to elicit its therapeutic effects. Moreover, Neuroimaging studies have demonstrated that acupuncture at PC6 can significantly modulate neural activity in multiple brain regions, including the middle frontal gyrus, precuneus, parahippocampal gyrus, superior temporal gyrus, fusiform gyrus, anterior cingulate cortex, and thalamus (50, 51). Different acupoints exert distinct effects on brain networks, whereas other points (e.g., HT7) predominantly influence mesolimbic pathways (52), Some studies have reported improved attention and cognitive performance in stroke patients after electroacupuncture at PC6 (53). These findings objectively support PC6’s efficacy in ‘awakening the spirit and enhancing intelligence’, suggesting that stimulating this acupoint can affect brain network nodes intimately linked to cognition. Building on this, our study takes a further step by applying non-invasive magnetic stimulation to PC6, aiming to achieve similar or even stronger effects. Preliminary work with magnetic stimulation shows parallel effects, enhancing functional connectivity on fMRI and strengthening alpha-band synchrony between frontal and occipital cortices on EEG (54, 55). Therefore, if this study observes cognitive improvement in the MAG group accompanied by enhanced functional brain network connectivity, it will provide robust evidence supporting the hypothesis that acupoint magnetic stimulation enhances cognition by mediating plastic reorganization of brain networks.

This study also incorporates numerous methodological considerations aimed at enhancing the quality of evidence and the reliability of result interpretation. First, we employed a three-arm randomized controlled design, where all participants received conventional high-frequency rTMS targeting the L-DLPFC, with additional PC6 intervention introduced as a variable. This design ensures ethical compliance while attributing intergroup differences to the specific effects of either magnetic stimulation or acupuncture at PC6. Since rTMS has been demonstrated to have therapeutic benefits for PSCI (56, 57), we avoided exposing the control group to entirely ineffective treatments, thereby safeguarding patient welfare. However, it is important to acknowledge that this approach may reduce the significance of intergroup differences. To address this, we ensured sufficient statistical power through rigorous sample size estimation and balanced enrollment criteria. Second, regarding blinding procedures, we implemented both assessor and patient blinding to the greatest extent possible. Although acupuncture and magnetic stimulation differ in somatosensory perception, the sham stimulation group used a simulated coil placed over PC6 without delivering pulses, making it difficult for participants to discern whether they received actual magnetic stimulation. All clinical assessments were conducted by independent evaluators unaware of group assignments to minimize subjective bias.

Moreover, we specifically applied multimodal imaging techniques for objective efficacy evaluation. fNIRS data will be collected at three time points (baseline, immediately after the first session, and after the 15th session) to capture both acute and cumulative effects. The post-first-session scan detects rapid hemodynamic changes that reflect the immediate neuromodulatory impact of a single magnetic- or acupuncture-stimulus, consistent with rTMS-related long-term potentiation (LTP) or inhibition mechanisms (58). This immediate measurement also serves as an early indicator of whether the target brain regions respond to the intervention. The time point after 15 sessions evaluates the cumulative or sustained effects following the full course of treatment. Both neuromodulation and acupuncture may exhibit dose-dependent plasticity effects, where repeated stimulation over days or weeks leads to lasting reorganization of neural circuits and improvements in clinical outcomes (59, 60). Therefore, data collected at the end of the 15-session course can reveal long-term therapeutic effects and changes in brain networks that underlie sustained cognitive improvements. Most importantly, comparing immediate and long-term changes can distinguish transient physiological responses from enduring neuroplastic adaptations. Resting-state fMRI provides whole-brain network analysis, as well as the reconstruction of cognition-related pathways (e.g., hippocampus-posterior cingulate cortex) (61–63). If magnetic stimulation at the PC6 acupoint has a unique effect, we anticipate that fMRI will capture specific network reorganization patterns in the MAG group, such as enhanced connectivity within the DMN and between the DMN and SN, which aligns with mechanisms of post-stroke cognitive recovery. Furthermore, we plan to correlate imaging metrics with cognitive scores to examine the relationship between brain network changes and clinical outcomes, thereby deepening the understanding of the underlying mechanisms.

## Limitations

This single-center exploratory trial has several limitations. Firstly, although the L-DLPFC was selected as the target region, target localization was implemented via cap-based approaches (the “5-cm rule”) rather than neuronavigation techniques. Previous studies have demonstrated the imprecision of the “5-cm rule” for rTMS stereotactic localization, with evidence showing that this method may result in stimulation over non-prefrontal regions in a substantial proportion of subjects (64–66). This cap-based approach may therefore induce inter-individual localization variability and potentially compromise the internal validity of the trial. Secondly, clinical heterogeneity in PSCI may still influence treatment response. While key factors such as stroke subtype, lesion characteristics, and time from stroke onset to enrollment will be documented and explored as covariates in multivariable and sensitivity analyses, the trial may be not powered for definitive subgroup analyses. Thirdly, the use of a single acupoint (PC6, Neiguan) enhanced the rigor of experimental control, yet it deviates from routine clinical practice where multi-acupoint acupuncture is the standard modality. Thus, extrapolation of the study results to real-world settings should be performed with caution. Fourthly, despite efforts to optimize masking, blinding may be imperfect due to the distinct somatosensory experiences between acupuncture and magnetic stimulation, which may introduce expectation-related bias. Finally, the follow-up duration is relatively short, which limits conclusions regarding the long-term durability of cognitive and network-level changes; larger multicenter trials with longer follow-up will be necessary to confirm sustained clinical benefits.

## Conclusion

This trial will provide the first RCT evidence on the cognitive benefits and network-level mechanisms of combining PC6 magnetic stimulation with standard left DLPFC rTMS for PSCI, using fNIRS–fMRI. Positive results would support a practical, non-pharmacological rehabilitation strategy and inform subsequent large-scale, multicentre trials in acupoint-based neuromodulation.
